# Nature-based activities improve the well-being of older adults

**DOI:** 10.1038/s41598-020-74828-w

**Published:** 2020-10-23

**Authors:** Angelia Sia, Wilson W. S. Tam, Anna Fogel, Ee Heok Kua, Kenneth Khoo, Roger C. M. Ho

**Affiliations:** 1 Centre for Urban Greenery and Ecology Research, National Parks Board, Singapore, 259569 Singapore; 2 Alice Lee Centre for Nursing Studies, Yong Loo Lin School of Medicine, National University of Singapore, Level 2, Clinical Research Centre, Block MD11, 10 Medical Drive, Singapore, 1175974 Singapore; 3 Brenner Centre for Molecular Medicine, Singapore Institute for Clinical Sciences, 30 Medical Drive, Singapore, 117609 Singapore; 4 Department of Psychological Medicine, National University of Singapore, Singapore, 119228 Singapore

**Keywords:** Environmental social sciences, Health care

## Abstract

Current literature shows that interaction with urban greenery can have a wide range of positive health outcomes. Targeted nature-based programs, such as therapeutic horticulture, have been shown to result in multiple health benefits for older adults residing in temperate environments, but much less research has been carried out on populations with different phenotypes, such as older Asian adults in the tropics. The current study investigated the effects of a 24-session therapeutic horticulture program on 47 older participants in Singapore, with an experimental pretest posttest design. We found that participants maintained healthy sleep patterns and psychological health, as well as showed reduced anxiety and improved cognitive functioning (p < 0.05). In addition, they reported an increase in mean happiness score after each session. This study provides new evidence using a comprehensive set of indicators across the affective, cognitive, functional, psychosocial and physical domains, supporting current literature on the benefits of nature programs, with a novel focus on tropical environments. It provides evidence that the nature-based intervention has the potential to be translated to programs to benefit older adults in the tropics.

## Introduction

Highly urbanised environments offer productivity and convenience, but they also contain more stressors that affect health negatively, such as higher heat load, carbon emmissions and pollution, compared to the rural environments^1^. The positive association between contact with urban nature and health has been well established in literature. Back in the 1860s, city planners were already pronouncing that nature both tranquilizes and enlivens the mind; reinvigorating one’s whole system^2^. The number of scientific manuscripts published on the subject has also increased exponentially in the recent years, reporting wide-ranging benefits, from lower mortality^3^ and morbidity^4^ to improvements in physical and mental health^5^. Findings from the meta-analyses show statistically significant associations with a wide range of health benefits, including reduced diastolic blood pressure, heart rate, salivary cortisol, incidence of type II diabetes and stroke, cardiovascular and all-cause mortality, as well as health-denoting associations with pregnancy outcomes, HRV, and HDL cholesterol, and self-reported improvements in health^6^. However, most of the research was conducted in temperate regions, offering very limited understanding on the health effects of nature exposure in regions with different climatic and environmental factors, such as the tropics^7^.

Although the benefits of nature exposure on health are well established, the specific mechanisms and pathways are still not well understood. One of the leading theories is the Biophilia Hypothesis, which proposes that love for nature and ability to thrive in a natural environment are inherent to humans, dictated by evolutionary drives and instincts^8^. In contrast, biophobia, the affiliation with technology and human artifacts, is deemed to be culturally acquired rather than inborn, and as such unnatural^9^. Consequently, the healing properties of nature exposure are thought to be due to returning to our basic instincts and natural predispositions. Another commonly cited theory is the Attention Restoration Theory (ART), which proposes that contact with nature provides humans a softly fascinating environment that allows the mind to restore attentional fatigue. Nature enables people to be engaged in an “effortless” state of mind, as compared to other physical environments, which demand directed attention and hence exhaust our cognitive resources^10^.

The benefits of contact with nature are particularly well documented for older adults^11^. One nature-based activity that is known to be popular with older adults is gardening. It has been shown to promote overall health and quality of life, physical strength, fitness and flexibility, cognitive ability, and socialization^12^. A treatment related to gardening is therapeutic horticulture (TH). This is a facilitated process through which participants enhance their well-being by being involved in plant and plant-related activities. Due to its documented benefits, TH is gaining importance and TH programs are found in a wide variety of healthcare, rehabilitative, and residential settings in North America and a few other temperate countries such as the United Kingdom and Japan. A systematic review on the effects of TH showed that there was pre-post improvement in well-being, anxiety and depression, social relations, cognitive and functional outcomes^13^. Of the 20 studies included in the review, 16 were conducted on participants from long-term residential care facilities, and as such the results cannot be generalized to communities or day-care settings^13^. Only one study was conducted in the tropical environment, where the study population was healthy older adults with good cognitive functioning and good mobility^14^. Therefore, whether TH is effective for the general older adult population residing in the tropics, and its impact on a range of physical and cognitive status measures requires further investigation. Moreover, long-term outcomes of TH on physical and mental health are largely unexplored.

The current study investigated the long-term effects of a 24 session (6-month) TH program on older adults (*n* = 47) from three senior day-care centers. The study aimed to provide a better understanding of TH programming in the tropical environment and its health outcomes among older adults in Singapore. The TH program aims to promote enhanced health and wellbeing. We hypothesized that participants would, over time, show improvements in perceived physical health, sleep hygiene, depression, anxiety, cognitive performance (orientation, attention, memory, language, spatial visualization and motor skill), and social connectedness. We also hypothesized that the program would improve momentary positive affect immediately after each session.

## Methods

### Participants

Elderly participants (n = 47, 33 females) with a mean age of 77.5 years (SD = 7.8, range 60–95) were recruited progressively from three senior day care centers (SAC) in Singapore, between May 2017 to December 2018. 12 participants were first recruited from SAC 1 and assigned to group 1. They received the intervention from May to October 2017. This was followed by group 2 (intervention from November 2017 to May 2018) which comprised another 7 participants from SAC 1, and group 3 (intervention from June to December 2018) which comprised 11 participants from SAC 2, and group 4 (intervention from January to July 2019) which comprised 9 participants from SAC 3, and lastly group 5 (intervention from January to July 2019 and carried out on days different from group 4) which comprised 8 participants from SAC 3. All participants from SAC 1 (n = 19, 14 females), SAC 2 (n = 11, 8 females) and SAC 3 (n = 17, 11 females) gave consent to participate in the Mini Mental State Exam (MMSE)^15^ to test for signs of dementia. An age and education adjusted formula MMSEadj = Raw MMSE − (0.471 × [education − 12]) + (0.131 × [age − 70]) was used to adjust the MMSE scores for those older than 75 years old^16^. We used the cut-off score of 17 and lower as an exclusion criterion, as an indicator of severe cognitive impairment^17^. Those excluded were deemed to benefit from a more individualized horticultural therapy treatment program. The distribution of age and years of education was balanced in the three SACs. The study was approved by the National Healthcare Group Domain Specific Review Board (DSRB), with the reference L2016/00949. The research was performed in accordance with relevant guidelines and regulations. All participants provided informed consent.

### Intervention

The intervention was a 24-session weekly TH program comprising an equal mix of horticultural-based and nature art activities. The program was developed with the input of a horticultural therapy expert registered with the American Horticultural Therapy Association (AHTA). In addition, we incorporated knowledge of local plants and related activities. The one-hour sessions followed a planned order, beginning with a series of introductory horticultural-based activities—growing pea sprouts, setting up planters, growing vegetables from different modes. This was alternated with a few sessions of simple nature-art activities (e.g. making sun-catchers, leaf sketching) before another few sessions of horticultural-based activities. The program is outlined in Table 1. All the sessions are designed to stimulate engagement of participants’ senses through touching, seeing and smelling the plant materials. While nature-art activities promote a sense of creativity and achievement, offering immediate gratification, horticultural-based activities encourage a waiting and nurturing attitude, offering a deep sense of purpose in the longer term. Hence, the combination of the two types of activities enabled the participants to experience complementary benefits throughout the program. The team of 4 who facilitated the program comprised personnel with experience in conducting urban farming workshops for older adults and they have received training by the AHTA registered horticultural therapy expert.

**Table 1:** Activities in the 24-week therapeutic horticulture intervention.

Week	Activity	Type	Week	Activity	Type
1	Growing pea sprouts	H	13	Terracotta pot painting	A
2	Setting up planters for herbs	H	14	Leaf printing	A
3	Growing vegetables from seeds	H	15	Propagating plants with stem cuttings	H
4	Growing vegetable plugs	H	16	Rock art	A
5	Making sun-catchers	A	17	Making origami flowers and body scrub	A
6	Sketching leaves	A	18	Plant Pruning	H
7	Harvesting vegetables	H	19	Planting succulents in bottles	H
8	Nature art collage	A	20	Making pandan rose	A
9	Planting wheat grass	H	21	Floral arrangement	A
10	Harvesting wheatgrass for juicing	H	22	Making potpourri	A
11	Making enzyme from plants	A	23	Making terrariums	H
12	Compost making	H	24	Vegetable printing on tote bags	A

“H” refers to horticultural-based activity and “A” refers to nature-art activity.

The sessions were carried out in a public garden, the Therapeutic Garden @HortPark, in five separate groups, with a mean group size of eight seniors (SD = 2.4, range 7–12). The garden has trees that provide shade, water features that soothe and calm, and plants that stimulate the sense of hearing, sight, touch and smell. Overall, the site was designed to bring a feeling of peace and wellbeing to its users. In addition, there are features such as customized raised planters to facilitate gardening by elderly participants. Throughout the intervention, the participants were provided transport in a minibus from their respective SAC to the Therapeutic Garden @HortPark. Each session followed an organized structure, comprising simple stretching exercises, introduction to the activity, hands-on activity and a conclusion phase where the participants shared their thoughts of the session.

### Outcome measures

#### Affective outcome

To evaluate affect before and after the intervention throughout the 24-week program, we used the simple Visual Analogue Scale (VAS), originally developed by Hayes and Patterson^18^. We used VAS to measure momentary positive affect as its ease of administration did not further burden the respondents. VAS before and after each session helped capture the momentary change in affect in the 24-month long study, where external changes surrounding participants may act as confounding factors. The happiness level was measured by asking participants to place a single vertical mark on a horizontal line at a point corresponding to their current subjective degree of happiness. The VAS, with the word "unhappiest" indicated on the left end and on the word "happiest" on the right end, is a simple and useful method to evaluate degree of subjective happiness in the elderly people^19^. Higher scores indicate a happier state. The scale has been tested to be a valid and reliable single item happiness instrument that is suitable for a wide range of respondents, including those illiterate^20^. VAS was introduced in the study after the first group (n = 12) completed intervention on the remaining 35 participants.

#### Cognitive outcomes

To evaluate the course of cognitive changes in participants over time, as well as to document their response to the treatment program, we used the MMSE^21^. As the majority of participants were above 70 years old, the Clinical Dementia Rating (CDR), which characterizes cognitive and functional performance in the domains of memory, orientation, judgment & problem solving, community affairs, home & hobbies, and personal care^22^, was used to provide an assessment of whether any of them developed dementia during the 24 month period. We had planned to notify the center manager concerned for follow up should the CDR score indicates mild dementia, but all participants maintained CDR scores within the healthy range throughout.

#### Functional outcomes

Sleep hygiene was assessed using Pittsburgh Sleep Quality Index (PSQI)^23^. The PSQI, a self-rated questionnaire, is a valid and reliable instrument for assessing sleep quality and disturbances in the non-clinical population^24^. It has 19 items in seven “component” scores: subjective sleep quality, sleep latency, sleep duration, habitual sleep efficiency, sleep disturbances, use of sleeping medication, and daytime dysfunction.

To measure participants’ functional ability in their basic activities of daily living (ADL), we used the Barthel Index (BI)^25^, which is commonly used to assess behaviours relating to everyday activities of post-stroke patients or those with other disabilities. Participants rated their ability on ten ADL functions, including feeding, personal hygiene and toilet use. Lower scores indicate less independence, whereas higher scores indicate greater independence. The BI is a valid and reliable instrument that has been recommended as the standard approach to assess physical disability^26^.

#### Psychosocial and Physical outcomes

To evaluate participants’ psychosocial health, we used the Zung Self-Rating Depression Scale (SDS)^27^ and the Zung Self-Rating Depression Scale (SAS)^28^ respectively. The SDS is a 20-item questionnaire that examines the affective, psychological and somatic symptoms associated with depression. The SAS questionnaire, on the other hand, has 20 items that measure anxiety levels, detecting cognitive, autonomic, motor and central nervous system symptoms. Each item is scored on a Likert scale ranging from 1 to 4, and the total is termed the raw score. Both the SDS and SAS have been shown to be valid and reliable instruments for screening anxiety and depression^29^.

To evaluate if participants were socially isolated, we used the five-item Friendship Scale (FS). The FS has been shown to be a valid and reliable tool to assess degree of social connectedness and loneliness^30^. Subjects who are very socially isolated will obtain scores in the range 0–11, those who are isolated or having little social support will obtain scores of 12– 15, those with some social support will obtain score of 16–18, and those who are socially connected range will obtain 19–21. The very socially connected will score within the range 22–24^30^.

Participants’ self-perceived health states were evaluated using the EQ-5D-3L visual analogue scale (EQ VAS). They rated their health on the vertical scale where the endpoints are labelled ‘Best imaginable health state’ and ‘Worst imaginable health state’. The EQ VAS has been found to be good scale for assessing older adults, with adequate acceptability and feasibility^31^. To compare the findings with previous studies from other populations, the results were additionally analysed across different age groups.

#### Time-points of measurement

**Figure 1:**
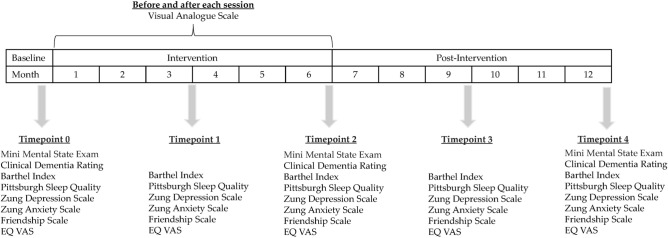
Outcome measures at different time points.

To understand the changes in participants’ functional, psycho-social and physical domains before, during and after the TH intervention, the respective outcome variables measured at baseline (up to two weeks before intervention) were compared with outcomes at 3 months (mid- intervention), 6 months (within one week post- intervention), 9 months (3 months post intervention) and 12 months (6 months post intervention). The specific time-points were chosen to assess dose–response relationship with TH exposure (3 months), and the long-term effects (9 and 12 months) to inform future public health recommendations. As cognitive impairments progress slowly, it is important to measure outcomes after a prolonged period, here 12 months. To understand the effect of each activity on participants’ momentary affect, the VAS was administered one hour before the start of each session at the respective SAC, and immediately after the session ended in the garden. The time points for the various measurements are illustrated in Fig. 1.

### Statistical analysis

We used the ANOVA repeated measures to compare the group means at the different time points, and Greenhouse–Geisser correction was applied where homogeneity of variance assumption was violated. Bonferroni corrected means were reported for the post-hoc comparisons. The paired-samples t-test was used to determine whether there was a statistically significant mean difference in individual VAS scores of happiness level before and after each session. All analyses were performed using IBM SPSS Statistics version 26. Alpha level of 0.05 was used as a level of statistical significance. Due to follow-up issues after the completion of the intervention, the responses at timepoints 3 (9-month) and 4 (12-month) dropped to 38 and 44 respectively. The cases with missing values were omitted in the pairwise comparisons.

## Results

### Sample characteristics

The general baseline characteristics of the participants are summarized in Table 2. The majority were females above 70 years old, of Chinese ethnicity and retired. The sample varied in socio-economic status, as indicated by housing type and years of schooling. Six participants required assistance to move about during the intervention, either in the form of wheelchair or walking aids.

**Table 2:** Baseline characteristics of the study participants.

Variable	Total		Participants					
			Age 60–70		Age 71–80		Age 81–95	
	N	%	N	%	N	%	N	%
**Total**	47	(100)	7	(14.9)	21	(44.7)	19	(40.4)
**Gender**								
Female	33	(70.2)	5	(71.4)	15	(71.4)	13	(68.4)
Male	14	(29.8)	2	(28.6)	6	(28.6)	6	(31.6)
**Ethnicity**								
Chinese	42	(89.4)	4	(57.1)	21	(100)	17	(89.5)
Indian	4	(8.5)	3	(42.9)	0	(0.0)	1	(5.3)
Malay	1	(2.1)	0	(0.0)	0	(0.0)	1	(5.3)
**Religion**								
Buddhist	18	(38.3)	3	(42.9)	11	(52.4)	4	(21.1)
Christian	10	(21.3)	1	(14.3)	3	(14.3)	6	(31.6)
Hindu	3	(6.4)	2	(28.6)	0	(0.0)	1	(5.3)
Islam	1	(2.1)	0	(0.0)	0	(0.0)	1	(5.3)
Taoist	10	(21.3)	1	(14.3)	4	(19)	5	(26.3)
Others	5	(10.6)	0	(0.0)	3	(14.3)	2	(10.5)
**Years of schooling**								
0	13	(27.7)	0	(0.0)	8	(38.1)	5	(26.3)
1–6	15	(31.9)	1	(14.3)	6	(28.6)	8	(42.1)
7–12	12	(25.5)	5	(71.4)	4	(19.0)	3	(15.8)
13–20	7	(14.9)	1	(14.3)	3	(14.3)	3	(15.8)
**Marital status**								
Divorced/separated	1	(2.1)	0	(0.0)	1	(4.8)	0	(0.0)
Married	19	(40.4)	5	(71.4)	10	(47.6)	4	(21.1)
Single	7	(14.9)	2	(28.6)	2	(9.5)	3	(15.8)
Widowed	20	(42.6)	0	(0.0)	8	(38.1)	12	(63.2)
**Living with**								
Alone	12	(27.7)	3	(42.9)	5	(23.8)	5	(26.3)
Daughter	6	(12.8)	0	(0.0)	4	(19.0)	2	(10.5)
Son	8	(17)	0	(0.0)	3	(14.3)	5	(26.3)
Spouse	12	(25.5)	0	(0.0)	5	(23.8)	3	(15.8)
With others	8	(17)	4	(57.1)	4	(19.0)	4	(21.1)
**Housing type**								
1–2 room	6	(12.8)	3	(42.9)	2	(9.5)	1	(5.3)
3 room	14	(29.8)	1	(14.3)	8	(38.1)	5	(26.3)
4–5 room	12	(25.5)	3	(42.9)	6	(28.6)	3	(15.8)
Condominium	1	(2.1)	0	(0.0)	0	(0.0)	1	(5.3)
Landed	5	(10.6)	0	(0.0)	3	(14.3)	2	(10.5)
Sheltered home	9	(19.1)	0	(0.0)	2	(9.5)	7	(36.8)

In Singapore, higher socio-economic status is linked with living in Landed property or Condominium (private estate). Other housing options are indicative of government housing.

### Effects of the intervention on participants’ momentary affect

We measured participants’ happiness level before and after each session using the VAS. 753 sets of pre-post responses from 35 participants were collected and analyzed. A score of “10” represents the happiest whilst “1” represents most unhappy. Post-session VAS scores was higher (M = 9.49, SD = 0.88) than pre-session scores (M = 8.88, SD = 1.34), a statistically significant increase of 0.60 [95% CI 0.53–0.67), t(752) = 16.32, *p* < 0.0005], with medium effect size (d = 0.61).

### Effects of the intervention on outcomes over time

We measured outcomes in the cognitive, functional psychosocial and physical domains at the timepoints illustrated in Fig. 1. Mean values at each time point and the results of statistical analyses for all the outcomes are summarised in Table 3.

**Table 3:** Summary of outcomes at the different timepoints and changes in outcomes over time.

Domain	Outcome measure	Baseline (n = 47)	Timepoint 1 3 months (n = 47)	Timepoint 2 6 months (n = 47)	Timepoint 3 9 months (n = 38)	Timepoint 4 12 months (n = 44)	F	p-value	Partial Eta-squared
Cognitive	MMSE (n = 44)	24.47 (4.007)	–	25.34 (3.835)	–	25.27 (3.878)	4.82	**.011**	.101
	CDR (n = 44)	.045 (.145)	–	.080 (.185)	–	.148 (.255)	4.226	**.027**	.089
Functional	BI (n = 38)	99.08 (2.561)	98.68 (3.618)	99.61 (1.794)	99.47 (1.942)	99.47 (1.942)	1.039	.377	.027
	PSQI (n = 38)	4.92 (2.318)	4.61 (2.308)	4.26 (2.321)	4.82 (2.228)	4.71 (2.205)	.881	.467	.023
Psycho-social & Physical	SDS (n = 38)	33.55 (7.738)	31.66 (7.099)	31.97 (7.618)	31.42 (7.024)	32.13 (8.201)	1.444	.227	.038
	SAS (n = 38)	29.11 (4.267)	29.42 (6.074)	26.92 (4.738)	27.24 (4.890)	28.00 (5.297)	4.269	**.007**	.103
	FS (n = 38)	19.37 (4.564)	18.24 (4.896)	19.11 (5.061)	19.26 (4.341)	19.71 (4.843)	1.719	.154	.044
	EQ VAS (n = 38)	74.08 (14.372)	78.37 (14.059)	75.39 (16.207)	76.97 (16.214)	75.92 (15.547)	.961	.417	.025

ANOVA was used to analyse differences over time (significant differences are in bold)

MMSE, Mini Mental State Exam, CDR, Clinical Dementia Rating, BI, Barthel Index, PSQI, Pittsburgh Sleep Quality Index, SDS, Zung Self-Rating Depression Scale, SAS, Zung Self-Rating Depression Scale, FS, Friendship Scale, EQ VAS, EQ-5D-3L Visual Analogue Scale.

### MMSE

The highest possible score for MMSE is 30, representing maximum cognitive performance. The results showed that there was statistically significant difference in mean MMSE score among the three time points measured. The TH treatment elicited a statistically significant increase of 0.87 in participants’ mean MMSE score at time point 2 compared to baseline, *p* = 0.01. At time-point 4, six months post treatment, there was a marginal drop of MMSE score by 0.07, but this was not statistically significant.

### CDR

The scoring of CDR ranges between 1 and 5. A score close to 0 indicates no dementia and a score of 1 indicates mild dementia. A higher score corresponds to an increasing severity in one’s cognitive decline^32^. The CDR was administered to assess if any participants had risks of dementia during the 24-month period. We had the intention of referring them for follow-up if needed. The results showed that there was a mild but statistically significant increase in the CDR scores over time, but overall, the scores were within the healthy range of close to 0.

### BI

The maximum score of 100 represents a fully independent subject capable of performing basic ADL whereas the lowest score of 0 represents a totally dependent state^27^. The results showed that the mean scores in BI were close to 100 at all the five timepoints. There was no significant difference in the scores among the timepoints.

### PSQI

All 7 questions are scored on a scale of 0–3, whereby 3 reflects the negative extreme. The maximum global score for PSQI is 21. A score of 5 or greater indicates a poor sleeper^33^. The results showed that the participants maintained healthy sleep quality at all the timepoints measured. They showed marginally better PSQI global scores compared to baseline during the intervention period at timepoints 2 and 3, but this was not significant (p > 0.05).

### SDS and SAS

Participants’ depression and anxiety states were assessed using SDS and SAS. The mean SDS and SAS scores at time points 0, 1, 2 3 and 4 are summarised in Table 3. They are below the cut-off points for clinical relevance of SDS^34^ and SDA^35^, which are 50 and 40 respectively. Higher scores indicate presence of depressive and anxiety disorders. The results showed that participants’ SDS and SAS scores were within the healthy range at all the five timepoints. There was marginal improvements (reduction) in mean SDS scores at the different timepoints compared to baseline but this was not significant. On the other hand, there was significant improvement in SAS scores across the time points. Compared to baseline, the TH intervention elicited significant improvements in mean SAS scores at timepoints 2 and 3, by − 2.18 ± 0.90 (*p* < 0.05) and − 1.87 ± 0.60 (*p* = 0.03).

### FS

Participants’ sense of social connectedness was assessed using the FS. There was no significant difference in mean FS scores among the different timepoints, and all the scores were equivalent to being socially connected.

### EQ VAS

There were no significant differences in the mean EQ VAS scores across the different time points. The mean scores for participants aged 65–74 and 75 and above are summarised in Table 4.

**Table 4:** Mean EQ VAS scores in participants of two age groups during and after intervention.

Age	Baseline	Timepoint 1 3 months during intervention	Timepoint 2 6 months during intervention	Timepoint 3 3 months post-intervention	Timepoint 4 6 months post-intervention
65–74 (n = 16)	75.63	81.1	78.1	77.7	79.4
75 + (n = 29)	73.10	74.5	75.2	77.7	73.9

## Discussion

The current study provided evidence in support of the existing literature on the effectiveness of TH in enhancing well-being of older adults, filling the information gap for the Asian phenotype in the tropical environment and providing novel information on the long-term effectiveness. The intervention resulted in several health benefits for the participants, namely more positive momentary affect, improved cognitive function and reduced anxiety. The effects of the latter two areas were sustained for up to 6 months after the intervention was completed. Throughout and six months after the intervention, the participants sustained their functional performance, maintaining healthy BI and PSQI scores. This is deemed to be a positive outcome, considering majority of them were aged 70 years and above, and would normally deteriorate in functional health in the span of one year. Similarly, they maintained a non-depressive state and a healthy ED VAS score. The mean EQ VAS scores for participants who were aged 65–74 years (n = 15) and 75 years and above (n = 29) during and after the intervention were consistently better that the population norm in the same age group in Thailand^36^ and the United Kingdom^37^. Overall, this study has demonstrated the positive effects of the 24-week TH on older adults in the tropics, in improving their momentary positive affect, cognitive performance and reduced anxiety over time.

Urban nature supports health by functioning as “salutogens”, factors in the environment that support health and well-being^38^, to promote positive experiences and behavior. Its benefits on the health and wellbeing of older adults have economic importance, considering the societal expenditures on health and related services for the group. Singapore, with 100% urban population, is expected to have more than 20% of its population over the age of 65 years by 2030. According to a recent survey "Understanding the Quality of Life of Seniors in Singapore", older adults in the country reported a lower quality of life than the general population in areas such as recreation, leisure and affect. We think that Singapore is well placed to introduce nature-based solutions to improve the quality of life of the older adult population, considering the profuse greenery and accessible green spaces managed throughout the entire island. Moreover, older adults have more hours of leisure time, and stand to derive deeper benefits by taking part in TH as a form of nature-based recreation and occupation.

There are several limitations to the study. For ethical reasons, a pretest posttest methodology was selected over a randomized control trial as the recruitment centres raised concerns about withholding the treatment from the potential control group. Additionally, budget constraints precluded adopting a waitlist randomized control trial approach. We were not able to include biological makers as objective assessments, as we had done in our past study^14^, for technical reasons linked to sample storage. The strength of this study was the long-term assessment of a lifestyle nature-based programme that measured several cognitive, executive function, lifestyle and behavioural outcomes in the often-overlooked population of older adults, with a relatively low drop-out rate.

## Conclusion

The current study evaluated the effects of a 24-week TH intervention programme on older adults conducted in the Therapeutic Garden @HortPark (Singapore), which has been specifically designed to support mental and physical health. The therapeutic programme was centred around nature exposure and caring for plants, a nurturing process that has been shown to promote physical and mental health, as well as development of a personal relationship with nature. The TH program promoted understanding and exploration of nature and cultivated a sense of purpose in the participants. In addition, we found that the participants maintained healthy sleep, psychological health and showed improved cognitive functioning over time. This study provides evidence that structured nature-based programmes play a positive role in maintaining and enhancing the physical and mental health and wellbeing in older populations. We conclude that the 24-week TH program is a promising intervention to be extended in other SACs in Singapore, to improve the wellbeing of older adults. We recommend that TH be introduced as a part of healthy aging recommendations in tropical climates and beyond.

## Data Availability

The data that support the findings of this study are available from the corresponding author, A.S., upon reasonable request.
