# Author Correction: Nature-based activities improve the well-being of older adults

**DOI:** 10.1038/s41598-021-96296-6

**Published:** 2021-08-16

**Authors:** Angelia Sia, Wilson W. S. Tam, Anna Fogel, Ee Heok Kua, Kenneth Khoo, Roger C. M. Ho

**Affiliations:** 1 Centre for Urban Greenery and Ecology Research, National Parks Board, Singapore, 259569 Singapore; 2 Alice Lee Centre for Nursing Studies, Yong Loo Lin School of Medicine, National University of Singapore, Level 2, Clinical Research Centre, Block MD11, 10 Medical Drive, Singapore, 1175974 Singapore; 3 Brenner Centre for Molecular Medicine, Singapore Institute for Clinical Sciences, 30 Medical Drive, Singapore, 117609 Singapore; 4 Department of Psychological Medicine, National University of Singapore, Singapore, 119228 Singapore

Correction to: *Scientific Reports*: 10.1038/s41598-020-74828-w, published online 23 October 2020

The original version of this Article contained an error in Affiliation 4, which was incorrectly given as ‘Department of Psychological Medicine, National University Hospital, Singapore, 119074, Singapore’. The correct affiliation is listed below:

Department of Psychological Medicine, National University of Singapore, Singapore, 119228, Singapore

In addition, the original version of this Article omitted an affiliation for Angelia Sia. The correct affiliations for Angelia Sia are listed below:

Centre for Urban Greenery and Ecology Research, National Parks Board, Singapore, 259569, Singapore

Department of Psychological Medicine, National University of Singapore, Singapore, 119228, Singapore

The original Article has been corrected.

